# Restrictive immigration policy and self-reported health among policy-vulnerable adults in the United States: A difference-in-differences study

**DOI:** 10.1016/j.jmh.2026.100434

**Published:** 2026-08-14

**Authors:** Sebastian O. Ekenze, Lorenza Arena, Jethro W. Zih-Shuo, Rabia Bega, Timothy M. Pawlik

**Affiliations:** Department of Surgery, The Ohio State University Wexner Medical Center and James Comprehensive Cancer Center, Columbus, OH, USA

**Keywords:** Immigration policy, Cancer survivorship, Health disparities, Difference-in-differences, Access to care

## Abstract

**Objective:**

We evaluated associations between implementation of Florida Senate Bill 1718 and self-reported physical and mental health among Hispanic and non-Hispanic Black adults, and examined whether these associations differed by cancer survivorship.

**Methods:**

Using 2022–2024 Behavioral Risk Factor Surveillance System data, Hispanic and non-Hispanic Black adults in Florida were compared with those in Southern control states; cancer survivors were propensity-matched 1:2 to adults without cancer. Survey-weighted difference-in-differences and triple-difference models estimated changes in self-reported poor physical and mental health days. Parallel pre-policy trends were assessed using event-study models on an extended 2020–2024 panel, and p values for state-comparative models were obtained using a wild cluster bootstrap.

**Results:**

Among 5991 respondents, the Florida-versus-control increase in poor physical health days among cancer survivors alone was imprecisely estimated (difference-in-differences, 4.97 days; 95% CI, −0.59 to 10.52; bootstrap p = 0.137). In the survey-weighted analysis, implementation of SB 1718 was associated with a differential increase of 7.09 physically unhealthy days per month among cancer survivors relative to adults without cancer (triple difference, 95% CI, 3.63–10.54). However, this estimate was substantially attenuated and no longer statistically significant in the unweighted analysis (1.34 days; 95% CI, −0.19 to 2.87). The estimate also varied when the largest survey weights were excluded or winsorized and was based on small Florida cancer-survivor samples. No robust differential association was identified for mentally unhealthy days.

**Conclusion:**

Although the survey-weighted analysis suggested an increase in physically unhealthy days among cancer survivors following implementation of Florida SB 1718, the magnitude and statistical significance of this estimate were not robust to the weighting specification. Small Florida cancer-survivor samples, unequal survey weights, and limited compositional comparability between Florida and the control states preclude a definitive conclusion regarding the policy’s association with self-reported health.

## 1 Introduction

Cancer survivorship in the United States has grown substantially, with >18 million individuals living with and after a cancer diagnosis, many experiencing persistent physical and psychosocial sequelae. (Wagle et al., 2025) In 2025, approximately 2.04 million new cancer cases and 618,120 cancer deaths were projected in the United States, with breast, prostate, lung, colorectal, and melanoma among the most commonly diagnosed cancers. (Siegel et al., 2025) Cancer burden varies substantially by race and ethnicity. Approximately 248,470 new cancer cases and 73,240 cancer deaths were projected among Black people in 2025, reflecting a disproportionately high cancer mortality burden. (Saka et al., 2025) Among Hispanic individuals, prostate and colorectal cancers are among the most commonly diagnosed cancers in men, while breast and uterine corpus cancers are among the most common in women; Hispanic individuals also experience comparatively high incidence of selected cancers, including liver, stomach, and cervical cancers. (American Cancer Society 2024) These disparities are shaped not only by clinical factors but also by structural determinants of health, including policies that influence access to care, economic stability, and social conditions. (Singh and Jemal, 2017; Yabroff et al., 2020 Feb 1) Public policy plays a central role in shaping health care access among cancer survivors, affecting insurance coverage, care-seeking behavior, and resource allocation. These effects may be more pronounced among immigrants, who often face language and cultural barriers and may avoid care due to concerns about legal status.

State and local policies play a pivotal role in shaping access to care for undocumented immigrants in the United States. In May 2023, Florida enacted Senate Bill 1718 (SB 1718), one of the most comprehensive state-level immigration measures in recent years; the law took effect July 1, 2023. (Senate Bill 1718 2023) SB 1718 introduced multiple provisions affecting immigrant communities, including mandatory E-Verify employment verification, restrictions on transporting undocumented individuals, and requirements that hospitals accepting Medicaid collect patient immigration status. (Senate Bill 1718 2023) Prior evidence suggests that restrictive immigration policies may reduce healthcare utilization, delay care, and increase psychological distress driven by fear of detention, deportation, and family separation. (Friedman and Venkataramani, 2021 Jul; Santos et al., 2025 Jan 9; The Lancet 2025 Jun 14) Such policy changes may discourage health care use because of fear of enforcement or administrative barriers, with effects extending beyond undocumented individuals to broader communities, including U.S.-born populations. (Friedman and Venkataramani, 2021 Jul; Liu et al., 2026 Jan 3) Despite the growing adoption of these policies, their associations with health outcomes, particularly among medically vulnerable groups such as cancer survivors, remain inadequately characterized.

Hispanic and non-Hispanic Black adults were considered policy-vulnerable groups because of potential direct or spillover exposure to restrictive immigration policies. Hispanic individuals constitute roughly 70%–75% of the undocumented U.S. population. (Baker and Warren, 2024; Liu et al., 2026 Jan 3) Nearly one in ten non-Hispanic Black individuals in the United States is foreign-born, including African and Caribbean immigrant communities that may also be affected by immigration-related policies. (Martinez and Passel, 2025) Restrictive immigration policies may additionally affect broader households and communities through fear, mistrust, reduced healthcare engagement, and structural barriers, including among individuals not directly subject to immigration enforcement. (Friedman and Venkataramani, 2021 Jul; Liu et al., 2026 Jan 3; Perreira and Pedroza, 2019 Apr 1) These mechanisms may adversely affect both physical and mental health, particularly among individuals requiring ongoing healthcare services. Despite these concerns, population-level evidence on the association between anti-immigration policies and health outcomes among cancer survivors, compared with individuals without a history of cancer, remains limited, particularly for Hispanic and non-Hispanic Black populations who bear a disproportionate cancer burden and persistent inequities in cancer care. (Singh and Jemal, 2017; Wagle et al., 2025; Yabroff et al., 2020 Feb 1) Using nationally representative Behavioral Risk Factor Surveillance System data from 2022 to 2024, we sought to characterize the association between Florida Senate Bill 1718 and self-reported physical and mental health among Hispanic and non-Hispanic Black adults with and without a history of cancer.

## 2 Materials and methods

### 2.1 Data source, study population, and cohort selection

A serial cross-sectional analysis was conducted using 2022–2024 Behavioral Risk Factor Surveillance System (BRFSS) data, a nationally representative, state-based survey of noninstitutionalized U.S. adults aged ≥18 years administered by state health departments in collaboration with the Centers for Disease Control and Prevention. (Behavioral Risk Factor Surveillance System 2026; CDC 2023; Hu et al., 2011 Mar 15) BRFSS employs complex sampling with stratification, clustering, and weighting to produce state- and national-level estimates. The study population comprised Hispanic and non-Hispanic Black adults, populations disproportionately affected by immigration-related policies, ^12,14^ including individuals with a history of cancer and matched non-cancer controls. To improve comparability, cancer survivors were matched 1:2 to non-cancer controls using nearest-neighbor propensity score matching with a caliper of 0.2. (Austin, 2011) Exact balance was enforced on sex and race/ethnicity; age differences were constrained within the specified caliper. (Austin, 2011) All cancer survivors were retained in the matched sample, and unmatched controls were excluded. Cancer survivorship status was evaluated as a prespecified subgroup characteristic to determine whether associations between SB 1718 and self-reported physical and mental health differed between cancer survivors and adults without a history of cancer. Because BRFSS does not collect information on immigration status, citizenship, or documentation status, analyses were conducted at the population-group level rather than according to individual immigration status. BRFSS data are publicly available and deidentified; this study was therefore exempt from The Ohio State University Institutional Review Board review. This study followed the Strengthening the Reporting of Observational Studies in Epidemiology (STROBE) reporting guideline. (Von Elm et al., 2007 Oct 16)

### 2.2 Exposures, outcomes and covariates

Primary exposures were cancer status, survey period relative to Florida Senate Bill 1718 (SB 1718), and state of residence among Hispanic and non-Hispanic Black adults with and without a history of cancer. Cancer status was defined by the BRFSS item: “Have you ever been told by a health professional that you had melanoma or any other type of cancer?” Respondents answering yes were classified as cancer survivors; individuals answering no were classified as non-cancer participants. The pre-policy period was defined as July 2022 through June 2023 and the post-policy period as August 2023 through December 2024. July 2023, the first month after SB 1718 took effect, was excluded as a transition month because policy exposure may have evolved around implementation rather than beginning instantaneously. (Liu et al., 2026 Jan 3) Hispanic and non-Hispanic Black adults residing in Florida comprised the intervention group; individuals residing in nearby southern states (Alabama, Arkansas, Georgia, Louisiana, Mississippi, North Carolina, South Carolina, and Tennessee) served as the primary control group. (Liu et al., 2026 Jan 3) Senate Bill 1718 is a Florida state law; therefore, Florida residence during the post-policy period was used to define exposure to the state policy environment, while Southern comparison states served as the counterfactual group. Southern comparison states were selected a priori based on geographic proximity within the Southern United States and the absence of exposure to SB 1718 during the study period. Florida and the comparison states differed substantially in racial/ethnic composition, which was addressed in a composition-balanced sensitivity analysis. In sensitivity analyses, the control group was expanded to include all other U.S. states. Because BRFSS does not collect immigration status, individual-level exposure to SB 1718 could not be measured directly. We therefore operationalized exposure as residence in Florida during the post-SB 1718 period among racial/ethnic groups plausibly vulnerable to direct or spillover effects of restrictive immigration policy environments. Hispanic adults were included because Hispanic individuals comprise the majority of the undocumented population in the United States. (Baker and Warren, 2024; Liu et al., 2026 Jan 3) Non-Hispanic Black adults were included because Black immigrant and immigrant-origin communities may also be affected by restrictive immigration climates and related spillover effects through household, community, health system, and structural pathways. (Martinez and Passel, 2025)

Primary outcomes were self-reported numbers of poor physical and poor mental health days in the past 30 days. Poor physical health days were measured by the BRFSS item: “For how many days during the past 30 days was your physical health not good?” Poor mental health days used the analogous item referencing stress, depression, and emotional problems. These measures are widely used in population health surveillance and have demonstrated validity and test–retest reliability. (Andresen et al., 2003; Moriarty et al., 2003)

Covariates included sociodemographic and geographic factors: age (18–64 vs ≥65 years), sex (female, male), race/ethnicity (non-Hispanic Black, Hispanic), educational attainment (less than high school, high school graduate, some college, college graduate or higher), employment status (employed, unemployed), marital status (married/partnered, unmarried, divorced/separated), household income (<$25,000; $25,000–$49,999; $50,000–$74,999; ≥$75,000), and rural residence (urban vs rural). (Social Determinants of Health 2026) In sensitivity analyses, non-Hispanic White cancer survivors and matched non-cancer adults were additionally included to assess robustness.

### 2.3 Statistical analysis

Descriptive estimates incorporated BRFSS sampling weights, strata, and primary sampling units. (CDC 2023) Regression models incorporated BRFSS survey weights, with standard errors clustered at the state level. Because the analytic sample was restricted to a propensity-matched subset and unmatched controls were excluded, these weighted model estimates are not interpreted as population-representative estimates and are described as survey-weighted model estimates from the matched analytic sample. Baseline characteristics were summarized with unweighted proportions (SMDs computed on the unweighted distributions); covariate balance was evaluated using standardized mean differences (SMDs), with SMD ≥0.10 indicating meaningful imbalance. (Austin and Stuart, 2015) Descriptive subgroup estimates were computed as domain (subpopulation) analyses, whereas the stratified regression models were fitted separately within each stratum. (Graubard and Korn, 1996) Policy-period associations were estimated using a quasi-experimental difference-in-differences (DiD) framework comparing Florida with Southern comparison states. (Wing et al., 2018) Difference-in-differences models included the Florida x policy-period interaction, state fixed effects, and interview month-year fixed effects; the Florida and policy-period main effects were absorbed by the corresponding fixed effects. Models were adjusted for age, sex, education, and employment, incorporated BRFSS survey weights, and used standard errors clustered at the state level. The coefficient for the Florida x policy-period interaction was the difference-in-differences estimand. Because BRFSS is a repeated cross-sectional survey, interview month-year fixed effects were included to account for secular temporal variation and seasonality across survey months rather than to model within-person changes over time.

Analyses were performed overall and stratified by cancer status, with cohort-specific DiD models for cancer survivors and matched non-cancer adults. To test whether associations varied by cancer status, Difference-in-difference-in-differences models additionally included cancer status and the corresponding lower-order interaction terms; the Florida x policy-period x cancer-status interaction was the difference-in-difference-in-differences estimand. Race- and ethnicity-specific heterogeneity was examined in stratified analyses among non-Hispanic Black and Hispanic adults. Cancer- and non-cancer-specific difference-in-differences estimates were obtained from models fit separately within each cancer-status stratum, whereas the difference-in-difference-in-differences was estimated from a single pooled model containing the Florida × policy period × cancer status interaction, with covariate and fixed-effect coefficients estimated jointly across cancer strata.

Additional sensitivity analyses included (1) expanding the comparison group to all other U.S. states and (2) within-Florida comparisons of the study population (non-Hispanic Black and Hispanic adults) with non-Hispanic White adults (non-Hispanic Black vs White; Hispanic vs White). To assess the parallel-trends assumption, we estimated event-study models using BRFSS data spanning 2020–2024. The 2021 survey periods were excluded from estimation because Florida contributed only 36 observations during January–June and none during July–December 2021; however, 2021 observations remained in the matching pool. The original 1:2 nearest-neighbor propensity-score matching procedure was repeated over the extended study window before restriction to the prespecified analytic population. Event-study models replaced the single post-policy interaction with half-year-specific interactions, with January–June 2023 designated as the reference period and July 2023 excluded as a transition period. The models otherwise retained the same covariates, survey weights, and state and interview month-year fixed effects as the primary models. For the difference-in-difference-in-differences analysis, we estimated half-year-specific interactions among Florida residence, cancer status, and survey period. Period-specific estimates and 95% confidence intervals were calculated using CR2 cluster-robust variance estimation with Satterthwaite degrees of freedom. Individual pre-policy coefficients were assessed using these confidence intervals, and the pre-policy coefficients were evaluated jointly using null-restricted wild cluster bootstrap tests with Webb six-point weights and 9999 replications, clustered by state. (Wing et al., 2018) Corresponding event-study models in the full unmatched analytic population were evaluated as sensitivity analyses. Because the primary state-comparative analyses included only nine state clusters, with Florida as the single treated state, p values for all DiD and DDD estimates were also calculated using null-restricted wild cluster bootstrap inference with Webb six-point weights, 9999 replications, and clustering by state. Conventional state-clustered and wild cluster bootstrap p values are reported together. In a sensitivity analysis addressing differences in measured sociodemographic composition, entropy-balancing weights were estimated to reweight control respondents to match Florida’s overall distribution of race/ethnicity, sex, education category, and age category. These balancing weights were combined with the original BRFSS survey weights, and the physical-health difference-in-difference-in-differences model was re-estimated in the composition-reweighted sample. Because uncertainty arising from estimation of the balancing weights was not incorporated, this analysis is reported as a point-estimate sensitivity analysis without a confidence interval or p value. To assess sensitivity to the survey-weighting specification, the physical-health DDD and cancer-survivor DiD models were also re-estimated without survey weights. As influence diagnostics, these models were re-estimated after excluding the five Florida cancer respondents with the largest survey weights and after winsorizing survey weights across the analytic sample at the 99th percentile. The exclusion and winsorization analyses are reported as point-estimate diagnostics. Estimates were reported as adjusted DiD and DDD parameters, expressed as absolute changes in number of days with 95% confidence intervals. Given the cross-sectional design, results were interpreted as associations rather than causal effects. All tests were 2-sided, with statistical significance defined as p < 0.05. Analyses were performed in R version 4.6.0 (R Foundation for Statistical Computing).

## 3 Results

### 3.1 Baseline cohort characteristics

Among 5991 non-Hispanic Black and Hispanic adults in the analytic sample, 1992 (33.2%) were cancer survivors and 3999 (66.8%) matched non-cancer controls; 971 (16.2%) resided in Florida and 5,020 (83.8%) in the Southern comparison states. The analytic sample was predominantly aged ≥65 years (n = 3830, 63.9%), female (n = 3653, 61.0%), and non-Hispanic Black (n = 5,042, 84.2%); Hispanic adults comprised 15.8% (n = 949) of the cohort. Baseline differences between Florida and the comparison states were observed for sex, race/ethnicity, and educational attainment (SMD > 0.10); other sociodemographic characteristics were broadly similar (SMD < 0.10) (Table 1). Baseline sociodemographic characteristics were well balanced between the pre-policy and post-policy periods, with no meaningful differences across age, sex, race/ethnicity, education, employment, income, or rural residence (all SMD < 0.10), supporting comparability over time (**Table S1**).

**Table 1:** Sociodemographic characteristics of Hispanic and non-Hispanic Black adults in the matched analytic sample, Florida versus Southern comparison states.

Socio-demographic characteristics n (unweighted %)	Overall (N = 5991)	Florida (N = 971)	Control States (N = 5020)	SMD
Survey Period	N = 5991	N = 971	N = 5020	0.002
Pre-Policy (July 2022 – June 2023)	2307 (38.5%)	373 (38.4%)	1934 (38.5%)	
Post-Policy (August 2023 – Dec 2024)	3684 (61.5%)	598 (61.6%)	3086 (61.5%)	
Age groups, n (unweighted %)	N = 5991	N = 971	N = 5020	0.066
18 – 64 years	2161 (36.1%)	376 (38.7%)	1785 (35.6%)	
≥65 years	3830 (63.9%)	595 (61.3%)	3235 (64.4%)	
*Sex at birth*	N = 5991	N = 971	N = 5020	0.150
Female	3653 (61.0%)	651 (67.0%)	3002 (59.8%)	
Male	2338 (39.0%)	320 (33.0%)	2018 (40.2%)	
*Race/Ethnicity*	N = 5991	N = 971	N = 5020	0.861
Non-Hispanic Black	5042 (84.2%)	499 (51.4%)	4543 (90.5%)	
Hispanic	949 (15.8%)	472 (48.6%)	477 (9.5%)	
*Education*	N = 5966	N = 962	N = 5004	0.118
Less than High school	757 (12.7%)	127 (13.2%)	630 (12.6%)	
High School Graduate	1862 (31.2%)	273 (28.4%)	1589 (31.8%)	
Some College	1568 (26.3%)	231 (24.0%)	1337 (26.7%)	
College graduate or higher	1779 (29.8%)	331 (34.4%)	1448 (28.9%)	
*Employment*	N = 1869	N = 342	N = 1527	0.050
Employed	1631 (87.3%)	303 (88.6%)	1328 (87.0%)	
Unemployed	238 (12.7%)	39 (11.4%)	199 (13.0%)	
*Marital status*	N = 5942	N = 964	N = 4978	0.099
Married/partnered	2346 (39.5%)	420 (43.6%)	1926 (38.7%)	
Unmarried	2292 (38.6%)	340 (35.3%)	1952 (39.2%)	
Divorced/separated	1304 (21.9%)	204 (21.2%)	1100 (22.1%)	
*Household income*	N = 4547	N = 714	N = 3833	0.091
<$25,000	1369 (30.1%)	196 (27.5%)	1173 (30.6%)	
$25,000–$49,999	1521 (33.5%)	235 (32.9%)	1286 (33.6%)	
$50,000–$74,999	670 (14.7%)	105 (14.7%)	565 (14.7%)	
$75,000+	987 (21.7%)	178 (24.9%)	809 (21.1%)	
*Rural Residence*	N = 5991	N = 971	N = 5020	0.058
Urban	5113 (85.3%)	845 (87.0%)	4268 (85.0%)	
Rural	878 (14.7%)	126 (13.0%)	752 (15.0%)	
				

SMD (Standardized mean difference) ≥ 0.10 indicate meaningful difference.

Control states = Southern comparison states: Alabama, Arkansas, Georgia, Louisiana, Mississippi, North Carolina, South Carolina, Tennessee.

### 3.2 Event-study and pre-policy trend assessment

In the extended event-study analyses, pre-policy coefficients varied across half-year periods. Joint wild cluster bootstrap tests did not reject the null of no differential pre-policy coefficients among cancer survivors, whereas differential pre-policy coefficients were detected among non-cancer respondents in several models. Covariate balance remained excellent in the extended matched cohort, with all absolute standardized mean differences of 0.01 or less (**Table S2**).

**Fig. 1:**
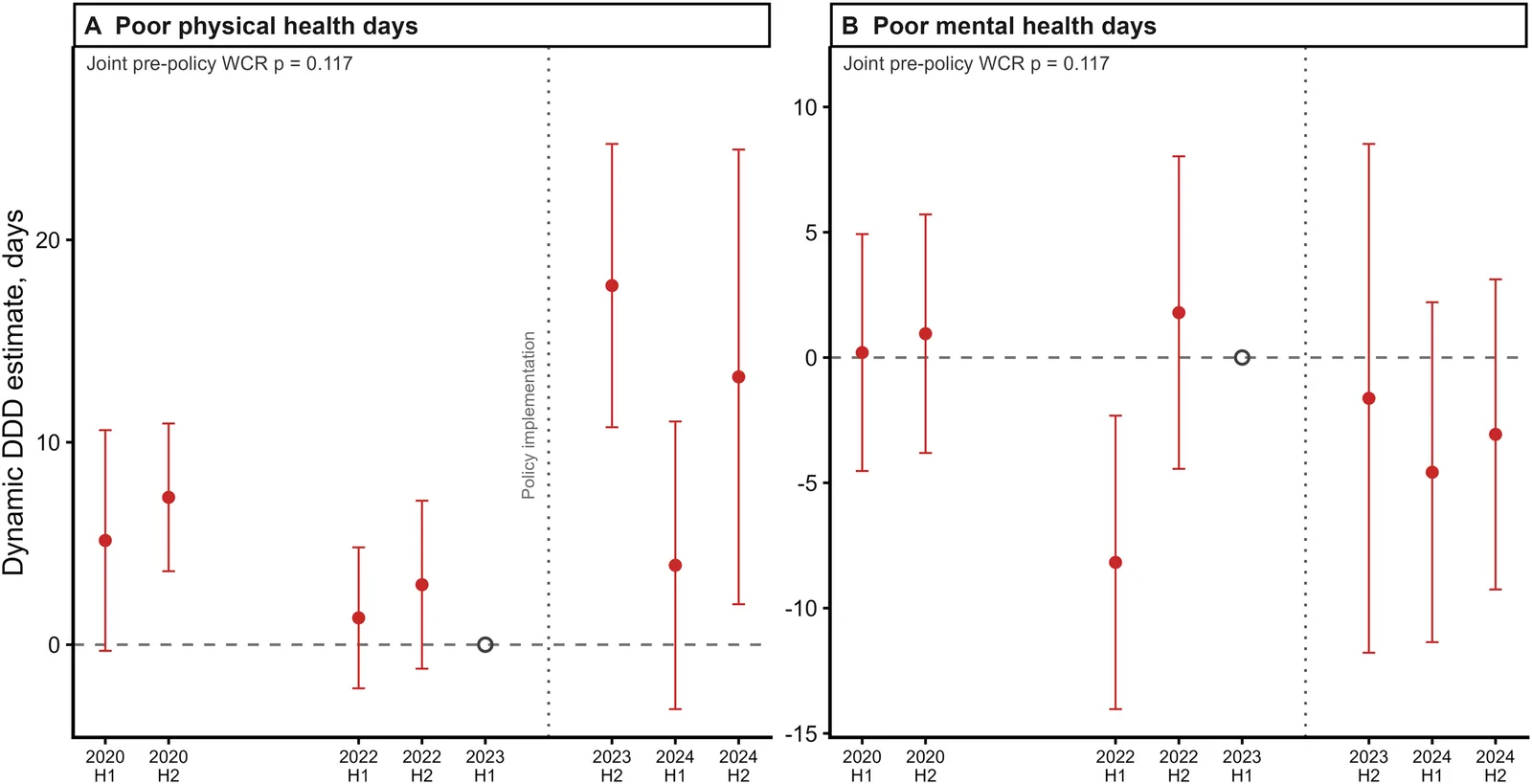
Dynamic triple-difference estimates before and after Florida SB 1718. Legend: Each point is the half-year-specific Florida x cancer status x survey period interaction coefficient (in days), estimated in the matched cohort of Hispanic and non-Hispanic Black adults in Florida and eight Southern comparison states, in which cancer survivors were 1:2 propensity-matched to non-cancer respondents using the original national matching procedure re-run over the extended 2020–2024 window. January-June 2023 is the reference period (open circle) and July 2023 was excluded as a transition period. Models were survey-weighted and adjusted for age category, sex, education, and employment, with state and interview month-year fixed effects. Error bars are 95% confidence intervals from CR2/Satterthwaite cluster-robust variance estimation clustered by state; the vertical dotted line marks policy implementation. The 2021 survey periods are absent because Florida contributed only 36 observations during January-June and none during July-December 2021. Pre-policy coefficients were evaluated jointly with a null-restricted wild cluster bootstrap; the joint pre-policy tests did not reject the null of no differential pre-policy triple-difference (physical p = 0.117; mental p = 0.117). Y-axis scales differ between panels. Panel A, poor physical health days; Panel B, poor mental health days.

In the matched cohort, the joint pre-policy tests for the dynamic difference-in-difference-in-differences coefficients did not reject the null for either poor physical health days (p = 0.117) or poor mental health days (p = 0.117); corresponding p-values in the full unmatched analytic population were 0.156 and 0.067, respectively (Fig. 1; **Figure S1; Table S3)**. Given the imprecision of the period-specific estimates and inference based on nine state clusters and a single treated state, these findings were not interpreted as confirmation of parallel trends.

### 3.3 Difference-in-differences estimates of outcomes between Florida and control states

Among all adults, self-reported poor physical health days increased in Florida from 3.63 to 5.50 days, compared with an increase from 3.83 to 4.31 days in the Southern control states; however, the differential change was not statistically significant (DiD, 1.11 days; 95% CI, −0.55 to 2.76; p = 0.228). Among cancer survivors, the estimate was in the same direction but imprecise (DiD, 4.97 days; 95% CI, −0.59 to 10.52; p = 0.118), whereas no differential change was observed among adults without cancer (DiD, −0.55 days; 95% CI, −1.79 to 0.70; p = 0.415). Poor mental health days decreased in Florida from 6.04 to 4.42 days while remaining relatively stable in the control states, from 5.33 to 5.38 days, with no statistically significant differential change overall (DiD, −0.76 days; 95% CI, −2.52 to 1.00; p = 0.423). Among cancer survivors, poor mental health days decreased in Florida relative to the control states (DiD, −3.46 days; 95% CI, −5.94 to −0.97; p = 0.026), whereas no differential change was observed among adults without cancer (DiD, −0.76 days; 95% CI, −1.91 to 0.40; p = 0.235) (Table 2).

**Table 2:** Adjusted difference-in-differences of poor physical and mental health days, Florida vs Southern controls.

Outcome	Florida		Southern controls		Adjusted DiD (95% CI), days	Cluster-robust p value
	Pre-policy	Post-policy	Pre-policy	Post-policy		
**Overall**						
Poor physical health days	3.63 (131)	5.50 (202)	3.83 (600)	4.31 (886)	1.11 (−0.55 to 2.76)	0.228
Poor mental health days	6.04 (130)	4.42 (202)	5.33 (606)	5.38 (900)	−0.76 (−2.52 to 1.00)	0.423
**Cancer**						
Poor physical health days	3.84 (46)	11.59 (61)	5.62 (179)	6.49 (239)	4.97 (−0.59 to 10.52)	0.118
Poor mental health days	8.22 (46)	4.06 (60)	6.50 (182)	5.06 (242)	−3.46 (−5.94 to −0.97)	0.026
**Non-cancer**						
Poor physical health days	3.56 (85)	3.21 (141)	3.14 (421)	3.51 (647)	−0.55 (−1.79 to 0.70)	0.415
Poor mental health days	5.30 (84)	4.56 (142)	4.90 (424)	5.50 (658)	−0.76 (−1.91 to 0.40)	0.235

Cell values are survey-weighted, unadjusted means, with unweighted Ns from the outcome-specific complete-case samples shown in parentheses. Adjusted DiD estimates were obtained from linear regression models including the Florida x policy-period interaction, state fixed effects, and interview month-year fixed effects, with BRFSS survey weights and standard errors clustered by state; the Florida and policy-period main effects were absorbed by the corresponding fixed effects. Models were adjusted for age, sex, education, and employment. Wild cluster bootstrap p values are reported in Supplementary Table 8. DiD, difference-in-differences; CI, confidence interval.

**Fig. 2:**
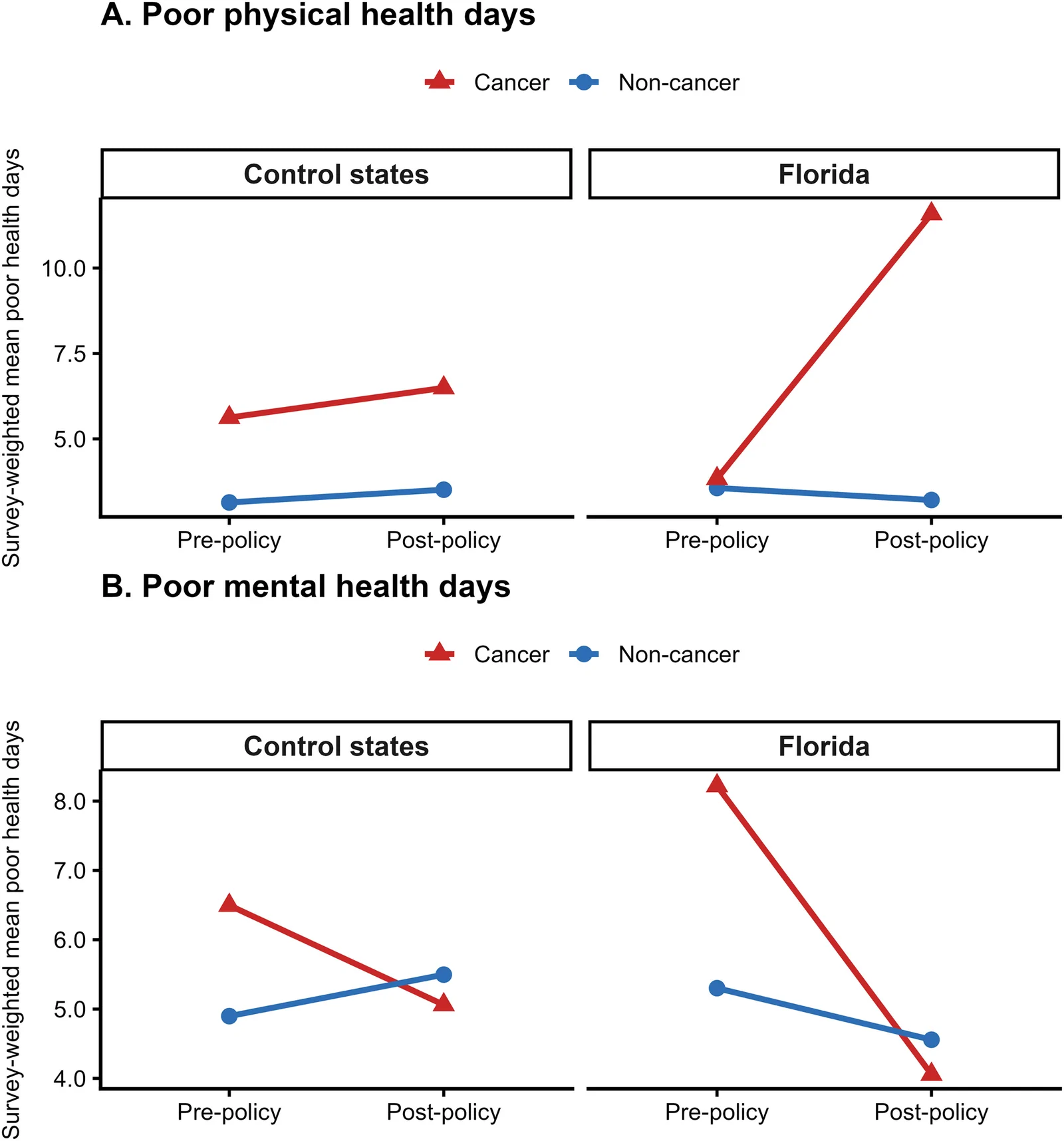
Survey-weighted mean poor physical and mental health days before and after Florida Senate Bill 1718, by cancer status and state group. Legend: Points are survey-weighted, unadjusted mean days of poor health in the past 30 days for the pre-policy (July 2022–June 2023) and post-policy (August 2023–December 2024) periods. Panel A, poor physical health days; Panel B, poor mental health days. Red triangles denote cancer survivors and blue circles denote adults without cancer; within each panel the left facet shows the eight Southern control states and the right facet shows Florida. These are the same survey-weighted cell means displayed in Table 2, Table 3. The adjusted difference-in-difference-in-differences estimate (Table 3) was obtained from a multivariable regression model incorporating covariates, state and interview month-year fixed effects, and survey weights, and is not equal to the simple triple difference calculated from the plotted means. Y-axis scales differ between panels. DDD, difference-in-difference-in-differences.

Among cancer survivors, poor physical health days increased from 3.84 to 11.59 in Florida and from 5.62 to 6.49 in the Southern control states; among adults without cancer, the corresponding changes were from 3.56 to 3.21 and from 3.14 to 3.51, respectively. These changes yielded an adjusted difference-in-difference-in-differences estimate of 7.09 days (95% CI, 3.63 to 10.54; p = 0.004) (Table 3; Fig. 2). In contrast, changes in poor mental health days did not differ between cancer survivors and adults without cancer across Florida and the control states (DDD, −1.32 days; 95% CI, −4.16 to 1.52; p = 0.389). Because the cancer- and non-cancer-specific difference-in-differences were estimated in separate stratified models, their arithmetic difference (5.52 days) need not equal the pooled triple-difference (7.09 days), which was estimated jointly in the full analytic sample and therefore has a different variance and covariance structure.

**Table 3:** Adjusted difference-in-difference-in-differences estimates by cancer status.

Group	Pre-policy	Post-policy	Adjusted DDD (95% CI), days	Cluster-robust p value
**Poor physical health days**				
Florida, cancer	3.84 (46)	11.59 (61)	7.09 (3.63 to 10.54)	0.004
Florida, non-cancer	3.56 (85)	3.21 (141)		
Southern controls, cancer	5.62 (179)	6.49 (239)		
Southern controls, non-cancer	3.14 (421)	3.51 (647)		
**Poor mental health days**				
Florida, cancer	8.22 (46)	4.06 (60)	−1.32 (−4.16 to 1.52)	0.389
Florida, non-cancer	5.30 (84)	4.56 (142)		
Southern controls, cancer	6.50 (182)	5.06 (242)		
Southern controls, non-cancer	4.90 (424)	5.50 (658)		

Cell values are survey-weighted, unadjusted means with unweighted Ns in parentheses. The adjusted DDD estimate and p value apply to each outcome and represent the cancer-versus-non-cancer contrast in the Florida-versus-control DiD, rather than estimates for the individual rows. Adjusted DDD estimates were obtained from linear regression incorporating the Florida × policy period × cancer status interaction, state and interview month-year fixed effects, survey weights, and standard errors clustered by state, with adjustment for age, sex, education, and employment. Because the regression additionally incorporates covariates and fixed effects, the adjusted DDD is not equivalent to the simple triple difference calculated from the displayed cell means. In addition, because the cancer- and non-cancer-specific DiDs reported in Table 2 were estimated from separate stratified models, the pooled DDD is not necessarily equal to their arithmetic difference. Southern control states were Alabama, Arkansas, Georgia, Louisiana, Mississippi, North Carolina, South Carolina, and Tennessee. Wild cluster bootstrap p values are reported in Supplementary Table 8. DDD, difference-in-difference-in-differences; DiD, difference-in-differences; CI, confidence interval.

Among non-Hispanic Black adults, poor physical health days increased in Florida relative to the control states (2.95 to 6.19 vs 3.60 to 3.95 days; DiD, 2.23 days; 95% CI, 0.96 to 3.51; p = 0.009). No differential change in poor physical health days was observed among Hispanic adults (DiD, −0.56 days; 95% CI, −6.94 to 5.83; p = 0.868). For mental health, Hispanic adults in Florida experienced a reduction in poor mental health days relative to the control states (6.23 to 4.47 vs 3.62 to 6.10 days; DiD, −5.60 days; 95% CI, −8.99 to −2.21; p = 0.012), whereas no differential change was observed among non-Hispanic Black adults (DiD, −1.02 days; 95% CI, −2.81 to 0.77; p = 0.297) (Table 4).

**Table 4:** Race/ethnicity-stratified adjusted difference-in-differences estimates, Florida vs Southern controls.

Outcome	Florida		Southern controls		Adjusted DiD (95% CI), days	Cluster-robust p value
	Pre-policy	Post-policy	Pre-policy	Post-policy		
**Non-Hispanic Black**						
Poor physical health days	2.95 (50)	6.19 (84)	3.60 (537)	3.95 (738)	2.23 (0.96 to 3.51)	0.009
Poor mental health days	5.58 (50)	4.30 (84)	5.60 (541)	5.21 (749)	−1.02 (−2.81 to 0.77)	0.297
**Hispanic**						
Poor physical health days	3.91 (81)	5.23 (118)	5.42 (63)	5.86 (148)	−0.56 (−6.94 to 5.83)	0.868
Poor mental health days	6.23 (80)	4.47 (118)	3.62 (65)	6.10 (151)	−5.60 (−8.99 to −2.21)	0.012

Analyses were conducted separately among non-Hispanic Black and Hispanic adults in the matched analytic sample. Cell values are survey-weighted, unadjusted means with unweighted Ns in parentheses. Adjusted DiD estimates were obtained using the same model specification as in Table 2. These race/ethnicity-stratified estimates are exploratory; because no formal interaction test comparing the Florida-by-policy-period associations across racial/ethnic groups was conducted, the estimates should not be interpreted as demonstrating differences in associations between racial/ethnic groups. Wild cluster bootstrap p values are reported in Supplementary Table 8. DiD, difference-in-differences; CI, confidence interval.

Expanding the comparison group to all other US states produced estimates in similar directions. Among all adults, poor physical health days increased in Florida relative to the comparison states (DiD, 1.41 days; 95% CI, 0.87 to 1.94; p < 0.001), while poor mental health days decreased (DiD, −1.13 days; 95% CI, −1.79 to −0.47; p = 0.001). Among cancer survivors, the differential increase in poor physical health days was larger (DiD, 8.26 days; 95% CI, 5.76 to 10.75; p < 0.001). Adults without cancer had a relative decrease in poor physical health days (DiD, −1.00 days; 95% CI, −1.43 to −0.58; p < 0.001) (**Table S4**).

### 3.4 Difference-in-differences estimates of outcomes: whites and study population within Florida

Within Florida, compared with non-Hispanic White adults, the combined study population (non-Hispanic Black and Hispanic adults) demonstrated no differential change in poor physical health days overall (DiD +0.91 days; 95% CI, −0.35 to 2.16; p = 0.157), but did exhibit a reduction in poor mental health days (DiD −1.67 days; 95% CI, −3.03 to −0.31; p = 0.016). Among cancer survivors, there was an increase in poor physical health days (DiD +4.85 days; 95% CI, 2.03 to 7.66; p = 0.001) and a reduction in poor mental health days (DiD −6.38 days; 95% CI, −9.02 to −3.73; p < 0.001) (**Table S5**).

### 3.5 Sensitivity analyses

Entropy balancing on race and ethnicity, sex, education, and age produced a physical-health DDD point estimate of 8.70 days. However, because an accompanying measure of uncertainty was not estimated, this analysis was considered descriptive and was not interpreted as corroborating the primary survey-weighted estimate. In the unweighted sensitivity analysis, the physical-health DDD estimate was 1.34 days (95% CI, −0.19 to 2.87), compared with 7.09 days (95% CI, 3.63 to 10.54) in the primary survey-weighted analysis. The corresponding survey-weighted point estimate was 4.74 days after excluding the five Florida cancer respondents with the largest survey weights and 6.03 days after winsorizing survey weights at the 99th percentile (**Table S6**). Race-specific within-Florida comparisons demonstrated an increase in poor physical health days for Hispanic cancer survivors versus non-Hispanic White cancer survivors (DiD +4.54 days; 95% CI, 1.50 to 7.57; p = 0.004) (**Table S7**). Under wild cluster bootstrap inference, the physical-health triple-difference remained statistically significant (bootstrap p = 0.032). In contrast, the reduction in poor mental health days among cancer survivors was not statistically significant (bootstrap p = 0.159). In exploratory race/ethnicity-stratified analyses, the increase in poor physical health days among non-Hispanic Black adults remained statistically significant (bootstrap p = 0.048), whereas the reduction in poor mental health days among Hispanic adults did not (bootstrap p = 0.079) (**Table S8**).

## 4 Discussion

In the primary survey-weighted analysis, Florida cancer survivors experienced a greater increase in self-reported poor physical health days compared with matched noncancer adults following policy implementation, while changes among cancer survivors alone did not reach statistical significance. However, the physical-health DDD was substantially attenuated and no longer statistically significant in the unweighted analysis (1.34 days; 95% CI, −0.19 to 2.87) and was based on small, unequally weighted Florida cancer samples. In contrast, poor mental health days declined among cancer survivors in Florida relative to control states, although this change did not differ significantly compared with noncancer adults. The race/ethnicity-stratified estimates were exploratory and did not follow a simple gradient of presumed policy exposure: the increase in poor physical health days was more apparent among non-Hispanic Black adults (DiD, +2.23 days; 95% CI, 0.96 to 3.51), whereas the reduction in poor mental health days was more apparent among Hispanic adults (DiD, −5.60 days; 95% CI, −8.99 to −2.21), a direction opposite to that hypothesized. Because the BRFSS does not measure immigration status or individual policy exposure, race and ethnicity serve only as group-level markers of policy vulnerability. Accordingly, these stratified estimates should be interpreted as exploratory heterogeneity rather than as an exposure gradient. Overall, the survey-weighted analysis suggested a possible differential increase in self-reported physical health burden among cancer survivors, but this association was not robust to the weighting specification.

The differential increase in poor physical health suggested by the primary survey-weighted analysis is notable because prior work on restrictive immigration policies has chiefly documented reduced health care utilization, poorer self-rated health, and worse mental health among Hispanic and immigrant populations, with limited evidence in medically complex groups and cancer survivorship. (Crookes et al., 2022; Friedman and Venkataramani, 2021 Jul; Santos et al., 2025 Jan 9) A recent Florida study likewise reported worsening physical health after SB 1718 among Hispanic adults overall, (Liu et al., 2026 Jan 3) and the direction of our survey-weighted estimate raises the possibility that cancer survivors may be particularly vulnerable, given the importance of surveillance, symptom management, and timely access to care. (El-Shami et al., 2015) However, the instability of the estimate across weighting specifications precludes determining whether the association was greater among cancer survivors. Survivors already face substantial access barriers; affordability, acceptability, and logistical obstacles have been associated with worse self-reported health and delayed care linked to higher emergency department use and mortality, especially among individuals with a cancer history. (Meernik et al., 2024; Singh and Jemal, 2017; Wagle et al., 2025; Yabroff et al., 2020 Feb 1) These patterns might be consistent with a possible mechanism whereby policies that increase fear, mistrust, or disengagement from health systems may be associated with unmet supportive care needs and physical symptom burden, even when eligibility for emergency services remains unchanged. (Crookes et al., 2022; Santos et al., 2025 Jan 9; The Lancet 2025 Jun 14) These findings highlight the need to better understand and address barriers to accessing ongoing cancer care among vulnerable populations.

The observed reduction in self-reported poor mental health days among cancer survivors warrants cautious interpretation. Prior studies have linked restrictive immigration policies to increased psychological distress, anxiety, and reduced care engagement, often attributed to fear of detention, family separation, and structural barriers. (Crookes et al., 2022; Friedman and Venkataramani, 2021 Jul; Martinez et al., 2015; Santos et al., 2025 Jan 9; The Lancet 2025 Jun 14) This reduction was opposite to that hypothesized and should not be interpreted as evidence that SB1718 improved mental health. Complementary, non-race-stratified event-study analyses showed variable pre-policy Florida-control differences in mental health, leaving open the possibility that pre-existing differences or other time-varying factors contributed to the observed mental-health estimates. These findings underscore the difficulty of assessing psychological responses to restrictive immigration policies using a general self-reported healthy-days measure and support further study using validated mental-health instruments and healthcare-utilization data. (Moriarty et al., 2003)

Exploratory race-stratified analyses suggested heterogeneity, with greater reported poor physical health among non-Hispanic Black adults. Structural racism has been identified as a root cause of racial health inequities. (Bailey et al., 2017; Bailey et al., 2021) Prior research has also documented poorer physical and functional well-being among Black cancer survivors. (Pinheiro et al., 2016) In contrast, the observed reductions in mental health among Hispanic adults differ from most prior immigration policy research and from a recent Florida study reporting worse mental health after SB 1718 among Hispanic adults overall, suggesting possible heterogeneity by race, cancer status, or measurement. (Crookes et al., 2022; Friedman and Venkataramani, 2021 Jul; Liu et al., 2026 Jan 3) Sensitivity analyses using alternative control groups and within-Florida comparisons with non-Hispanic White adults produced estimates in the same direction, suggesting that control-group selection alone may not explain the primary estimate. However, these analyses did not resolve its sensitivity to the weighting specification or the limitations arising from small, unequally weighted Florida cancer cells. The direction of these estimates is consistent with prior evidence associating restrictive immigration policies with differences in health outcomes, potentially related to reduced care engagement and structural barriers. (Crookes et al., 2022; Friedman and Venkataramani, 2021 Jul; Santos et al., 2025 Jan 9) In contrast, variability in mental health estimates across analyses underscores the need for cautious interpretation of self-reported mental health outcomes in this context. (Crookes et al., 2022; Friedman and Venkataramani, 2021 Jul; Santos et al., 2025 Jan 9)

These findings have implications for public health, oncology, and health policy. Restrictive immigration policies have been associated with reduced health care use and broader differences in health outcomes across communities, including US-born and naturalized individuals through shared household and community stressors. (Friedman and Venkataramani, 2021 Jul; Liu et al., 2026 Jan 3; Perreira and Pedroza, 2019 Apr 1; Santos et al., 2025 Jan 9) In this context, the higher reported poor physical health suggested by the survey-weighted analyses among Hispanic and non-Hispanic Black cancer survivors is notable, given the reliance of survivorship care on sustained access to follow-up, symptom management, and coordinated services. (Atlas et al., 2025; El-Shami et al., 2015; Hart et al., 2024; Yabroff et al., 2020 Feb 1) These findings highlight the need for policymakers and health systems to consider potential health implications of restrictive immigration policies for medically vulnerable populations. Approaches that reduce barriers to care and support continuity of survivorship services may help address these disparities. (Atlas et al., 2025; Hart et al., 2024; Kasherman et al., 2024; Mayer et al., 2017)

This study has several limitations. First, the BRFSS does not capture immigration status; thus, the study population included Hispanic and non-Hispanic Black adults regardless of immigration status, which may underestimate differences associated with SB 1718 among undocumented individuals. (Martinez et al., 2015) This approach may introduce exposure misclassification because Hispanic and non-Hispanic Black adults vary in immigration status, citizenship, nativity, socioeconomic position, healthcare access, and direct exposure to enforcement. Other potentially affected groups, including Arab, Muslim, Asian, and other immigrant-origin populations, could not be separately evaluated in this BRFSS-based analysis. Second, Florida and the comparison states differed substantially in racial/ethnic composition and, to a lesser extent, in sex and education. Although entropy balancing produced a point estimate similar in direction to the primary survey-weighted result, the absence of an accompanying measure of uncertainty precluded inferential interpretation. Moreover, statistical reweighting addressed only observed characteristics and could not fully resolve the limited compositional overlap, imbalance within small analytic subgroups, or unmeasured differences between Florida and the comparison states. Because the analysis was restricted to a propensity-matched subset and unmatched controls were excluded, the survey-weighted estimates should not be interpreted as population-representative. The physical-health estimate was also sensitive to the weighting specification and was based on small, unequally weighted Florida cancer cells, limiting its precision, stability, and generalizability. Third, outcomes were based on the CDC Healthy Days measures and therefore reflect self-reported rather than clinically assessed physical and mental health. Although these measures are widely used for population health surveillance, (Hennessy et al., 1994; Moriarty et al., 2003) they are subject to recall and reporting bias and may not fully capture symptom severity, specific clinical conditions, or differences in interpretation across populations. Fourth, although the design incorporated fixed effects and multiple comparison groups, residual confounding from unmeasured time-varying factors cannot be excluded. Post-pandemic recovery dynamics, labor market conditions, healthcare system disruptions, and state-specific political or economic trends during 2022–2024 may have influenced self-reported health outcomes and could partly account for estimated policy-associated changes. Fifth, the parallel-trends assumption cannot be definitively verified. Inference was limited by nine state clusters and a single treated state, and wild cluster bootstrap procedures do not eliminate the limitations associated with this design. Florida contributed insufficient observations for the 2021 event-study periods, while the extended pre-policy window included pandemic-period observations from 2020. Differential Florida-control pre-policy movement was identified among non-cancer respondents, whereas joint tests of the dynamic triple-difference pre-policy coefficients did not reject the null for the cancer-versus-non-cancer contrast underlying the primary estimate. These results were imprecise and are compatible with, but do not confirm, the identifying assumption for the difference-in-difference-in-differences analysis. Relatedly, policy exposure may also have begun before formal implementation through public debate, media coverage, and anticipatory behavioral responses, and the available post-policy period may not capture longer-term consequences. Finally, the cross-sectional design precluded assessment of individual-level changes and limits causal inference. Findings from a single-state policy may not be generalizable to other settings. Future studies should incorporate immigration status, longitudinal designs, validated clinical outcomes, and linked survey and administrative data to better define mechanisms and quantify associations in high-risk populations.

In conclusion, the survey-weighted model suggested an increase in physically unhealthy days among Florida cancer survivors following implementation of SB 1718. However, the estimate was substantially attenuated and no longer statistically significant in the unweighted analysis and was based on small, unequally weighted Florida samples. Together with limited compositional comparability between Florida and the control states, these findings preclude a robust conclusion regarding the association between SB 1718 and self-reported health among cancer survivors. These findings require confirmation in studies incorporating individual-level measures of immigration status and policy exposure, validated clinical outcomes, and longitudinal designs.

## 5 Disclosures

None

## Funding

No funds, grants, or other support was received

## Human ethics and consent to participate declaration

Not applicable

Ethics declaration

The author(s) declare(s) that the study does not involve humans nor animal subjects.

The authors have nothing to declare.

## Disclaimer

None.

## CRediT authorship contribution statement

**Sebastian O. Ekenze:** Writing – review & editing, Writing – original draft, Visualization, Validation, Software, Methodology, Formal analysis, Data curation, Conceptualization. **Lorenza Arena:** Writing – review & editing, Writing – original draft, Methodology, Conceptualization. **Jethro W. Zih-Shuo:** Writing – original draft, Visualization, Methodology. **Rabia Bega:** Writing – original draft, Methodology. **Timothy M. Pawlik:** Writing – review & editing, Validation, Supervision, Conceptualization.

## Declaration of competing interest

The authors declare no competing interests

## Appendix Supplementary materials

Image, application 1

Image, application 2
