# Supplementary material for: Restrictive immigration policy and self-reported health among policy-vulnerable adults in the United States: A difference-in-differences study
Source: J Migr Health. 2026 Aug 14;14:100434. doi: 10.1016/j.jmh.2026.100434 (PMC13520896; doi:10.1016/j.jmh.2026.100434)
Supplement: Supplementary file 1 [file mmc2.docx]

Supplementary Material

**Table S1. Baseline characteristics of the matched analytic sample before and after implementation of Florida Senate Bill 1718 (N = 5,991)**

| Socio-demographic characteristics  n (unweighted %) | Overall  (N=5,991) | Pre-policy  (Jul 2022–Jun 2023)  (N=2,307) | Post-policy  (Aug 2023–Dec 2024)  (N=3,684) | SMD |
| --- | --- | --- | --- | --- |
| *State group* | N=5991 | N=2307 | N=3684 | 0.002 |
| Southern comparison states | 5,020 (83.8%) | 1,934 (83.8%) | 3,086 (83.8%) |  |
| Florida | 971 (16.2%) | 373 (16.2%) | 598 (16.2%) |  |
| *Age groups* | N=5991 | N=2307 | N=3684 | 0.064 |
| 18 – 64 years | 2,161 (36.1%) | 876 (38.0%) | 1,285 (34.9%) |  |
| ≥65 years | 3,830 (63.9%) | 1,431 (62.0%) | 2,399 (65.1%) |  |
| *Sex at birth* | N=5991 | N=2307 | N=3684 | 0.013 |
| Female | 3,653 (61.0%) | 1,416 (61.4%) | 2,237 (60.7%) |  |
| Male | 2,338 (39.0%) | 891 (38.6%) | 1,447 (39.3%) |  |
| *Race/Ethnicity* | N=5991 | N=2307 | N=3684 | 0.075 |
| Non-Hispanic Black | 5,042 (84.2%) | 1,980 (85.8%) | 3,062 (83.1%) |  |
| Hispanic | 949 (15.8%) | 327 (14.2%) | 622 (16.9%) |  |
| *Education* | N=5966 | N=2301 | N=3665 | 0.051 |
| Less than High school | 757 (12.7%) | 300 (13.0%) | 457 (12.5%) |  |
| High School Graduate | 1,862 (31.2%) | 691 (30.0%) | 1,171 (32.0%) |  |
| Some College | 1,568 (26.3%) | 591 (25.7%) | 977 (26.7%) |  |
| College graduate or higher | 1,779 (29.8%) | 719 (31.2%) | 1,060 (28.9%) |  |
| *Employment* | N=1869 | N=746 | N=1123 | 0.073 |
| Employed | 1,631 (87.3%) | 640 (85.8%) | 991 (88.2%) |  |
| Unemployed | 238 (12.7%) | 106 (14.2%) | 132 (11.8%) |  |
| *Marital status* | N=5942 | N=2287 | N=3655 | 0.038 |
| Married/partnered | 2,346 (39.5%) | 903 (39.5%) | 1,443 (39.5%) |  |
| Unmarried | 2,292 (38.6%) | 860 (37.6%) | 1,432 (39.2%) |  |
| Divorced/separated | 1,304 (21.9%) | 524 (22.9%) | 780 (21.3%) |  |
| *Household income* | N=4547 | N=1757 | N=2790 | 0.044 |
| <$25,000 | 1,369 (30.1%) | 551 (31.4%) | 818 (29.3%) |  |
| $25,000–$49,999 | 1,521 (33.5%) | 568 (32.3%) | 953 (34.2%) |  |
| $50,000–$74,999 | 670 (14.7%) | 269 (15.3%) | 401 (14.4%) |  |
| $75,000+ | 987 (21.7%) | 369 (21.0%) | 618 (22.2%) |  |
| *Rural Residence* | N=5991 | N=2307 | N=3684 | 0.012 |
| Urban | 5,113 (85.3%) | 1,975 (85.6%) | 3,138 (85.2%) |  |
| Rural | 878 (14.7%) | 332 (14.4%) | 546 (14.8%) |  |
|  |  |  |  |  |

SMD (Standardized mean difference) ≥ 0.10 indicate meaningful difference

Control states = Southern comparison states: Alabama, Arkansas, Georgia, Louisiana, Mississippi, North Carolina, South Carolina, Tennessee

**Table S2.** Covariate balance in the extended matched cohort

| **Covariate** | **Overall** | **Florida** | **Southern control states** |
| --- | --- | --- | --- |
| Age category | −0.004 | −0.002 | −0.004 |
| Sex | −0.009 | −0.001 | −0.010 |
| Race/ethnicity | 0.001 | −0.001 | 0.000 |

Values are standardized mean differences comparing cancer survivors with non-cancer respondents in the extended matched cohort. The original national 1:2 nearest-neighbor propensity-matching procedure was repeated using the 2020–2024 matching pool, with age category included in the propensity score, exact matching on sex and race/ethnicity, and a caliper of 0.2. The matched sample was subsequently restricted to non-Hispanic Black and Hispanic adults in Florida and eight Southern control states (N=12,980; Florida, n=1,610). All absolute standardized mean differences were 0.01 or less.

**Table S3.** Joint pre-policy tests

| **Pre-policy joint test** | **Full analytic population, physical** | **Full analytic population, mental** | **Matched cohort, physical** | **Matched cohort, mental** |
| --- | --- | --- | --- | --- |
| Overall (pooled difference-in-differences) | 0.056 | 0.097 | 0.006 | 0.025 |
| Cancer-stratum difference-in-differences | 0.333 | 0.265 | 0.331 | 0.264 |
| Non-cancer-stratum difference-in-differences | 0.038 | 0.061 | 0.042 | 0.043 |
| Dynamic triple-difference (Florida × cancer status × period) | 0.156 | 0.067 | 0.117 | 0.117 |

Values are p values from null-restricted wild cluster bootstrap tests of the joint null that all differential pre-policy coefficients equal zero, using Webb six-point weights with 9,999 replications and clustering by state. Each test jointly evaluated four pre-policy periods. The matched cohort was used for the primary analysis; the full unmatched analytic population was evaluated as a sensitivity analysis.

**Table S4.** Sensitivity analysis using all other US states as the comparison group

| **Outcome** | **Florida** | | **Other US states** | | **Adjusted DiD (95% CI), days** | **Cluster-robust p value** |
| --- | --- | --- | --- | --- | --- | --- |
|  | **Pre-policy** | **Post-policy** | **Pre-policy** | **Post-policy** |  |  |
| **Overall** |  |  |  |  |  |  |
| Poor physical health days | 3.63 (131) | 5.50 (202) | 4.02 (4,335) | 4.25 (7,101) | 1.41 (0.87 to 1.94) | <0.001 |
| Poor mental health days | 6.04 (130) | 4.42 (202) | 4.90 (4,370) | 4.92 (7,172) | −1.13 (−1.79 to −0.47) | 0.001 |
| **Cancer** |  |  |  |  |  |  |
| Poor physical health days | 3.84 (46) | 11.59 (61) | 6.58 (1,294) | 6.71 (2,063) | 8.26 (5.76 to 10.75) | <0.001 |
| Poor mental health days | 8.22 (46) | 4.06 (60) | 5.62 (1,307) | 5.76 (2,082) | −3.25 (−5.11 to −1.39) | 0.001 |
| **Non-cancer** |  |  |  |  |  |  |
| Poor physical health days | 3.56 (85) | 3.21 (141) | 3.07 (3,041) | 3.34 (5,038) | −1.00 (−1.43 to −0.58) | <0.001 |
| Poor mental health days | 5.30 (84) | 4.56 (142) | 4.63 (3,063) | 4.61 (5,090) | −0.51 (−1.22 to 0.21) | 0.170 |

This sensitivity analysis expanded the comparison group to all other US states. Cell values are survey-weighted, unadjusted means with unweighted Ns in parentheses; Ns reflect the outcome-specific complete-case samples. Adjusted DiD estimates were obtained using the same model specification as in Table 2, with all other US states serving as the comparison group. Expanding the comparison group increased the number of control-state clusters but did not address the limitation of a single treated state. Wild cluster bootstrap p values are reported in Supplementary Table 8. DiD, difference-in-differences; CI, confidence interval.

**Table S5**. Within-Florida adjusted DiD: non-Hispanic Black and Hispanic adults vs non-Hispanic White adults

| **Outcome** | **Non-Hispanic Black and Hispanic adults** | | **Non-Hispanic White adults** | | **Adjusted DiD (95% CI), days** | **p value** |
| --- | --- | --- | --- | --- | --- | --- |
|  | **Pre-policy** | **Post-policy** | **Pre-policy** | **Post-policy** |  |  |
| **Overall** |  |  |  |  |  |  |
| Poor physical health days | 3.63 (131) | 5.50 (202) | 2.75 (1,297) | 3.62 (1,888) | 0.91 (−0.35 to 2.16) | 0.157 |
| Poor mental health days | 6.04 (130) | 4.42 (202) | 3.92 (1,304) | 5.01 (1,902) | −1.67 (−3.03 to −0.31) | 0.016 |
| **Cancer** |  |  |  |  |  |  |
| Poor physical health days | 3.84 (46) | 11.59 (61) | 3.84 (358) | 5.26 (526) | 4.85 (2.03 to 7.66) | 0.001 |
| Poor mental health days | 8.22 (46) | 4.06 (60) | 2.86 (366) | 5.91 (531) | −6.38 (−9.02 to −3.73) | <0.001 |

This within-Florida analysis compared non-Hispanic Black and Hispanic adults with non-Hispanic White adults. Cell values are survey-weighted, unadjusted means with unweighted Ns in parentheses, reflecting the outcome-specific complete-case sample. Adjusted DiD estimates are from linear regression incorporating the study-group-by-policy-period interaction and interview month-year fixed effects, with survey weights and adjustment for age, sex, education, and employment. Because the analysis was within a single state, state-level clustering and wild cluster bootstrap inference were not applicable. NHW, non-Hispanic White.

**Table S6.** Sensitivity of physical-health estimates to survey weighting, influential observations, and measured sociodemographic composition

| **Analysis** | **Physical-health DDD, days** | **Cancer-survivor physical-health DiD, days** |
| --- | --- | --- |
| Primary survey-weighted analysis | 7.09 (3.63 to 10.54) | 4.97 (−0.59 to 10.52) |
| Unweighted analysis | 1.34 (−0.19 to 2.87) | 0.51 (−1.19 to 2.20) |
| Excluding the five Florida cancer respondents with the largest survey weights | 4.74 | 3.23 |
| Survey weights winsorized at the 99th percentile | 6.03 | 4.20 |
| Measured-composition reweighted survey analysis | 8.70 | — |

Cluster-robust 95% CIs are shown for the primary and unweighted specifications. The exclusion and winsorization analyses were conducted as influence diagnostics and are presented as point estimates. In the measured-composition reweighted analysis, control observations were reweighted toward Florida's overall distribution of race/ethnicity, sex, education, and age while incorporating the original BRFSS survey weights. After reweighting, the standardized mean differences for these characteristics were reduced to approximately zero in the overall sample. The corresponding DDD is presented as a point estimate without a confidence interval because uncertainty arising from estimation of the additional balancing weights was not incorporated. The cancer-specific DiD was not estimated under this reweighting specification (—). DDD, difference-in-difference-in-differences; DiD, difference-in-differences; CI, confidence interval.

**Table S7**. Within-Florida race/ethnicity-specific adjusted DiD vs non-Hispanic White adults

| **Outcome** | **Comparison** | **Comparison-group unweighted N** | | **Non-Hispanic White unweighted N** | | **Adjusted DiD (95% CI), days** | **p value** |
| --- | --- | --- | --- | --- | --- | --- | --- |
|  |  | **Pre-policy** | **Post-policy** | **Pre-policy** | **Post-policy** |  |  |
| Poor physical health days | NHB vs NHW, all adults | 50 | 84 | 1,297 | 1,888 | 1.68 (−0.21 to 3.58) | 0.082 |
| Poor physical health days | Hispanic vs NHW, all adults | 81 | 118 | 1,297 | 1,888 | 0.88 (−0.53 to 2.29) | 0.222 |
| Poor physical health days | NHB vs NHW, cancer | 16 | 26 | 358 | 526 | 4.23 (−0.49 to 8.95) | 0.079 |
| Poor physical health days | Hispanic vs NHW, cancer | 30 | 35 | 358 | 526 | 4.54 (1.50 to 7.57) | 0.004 |
| Poor mental health days | NHB vs NHW, all adults | 50 | 84 | 1,304 | 1,902 | −2.59 (−4.69 to −0.49) | 0.016 |
| Poor mental health days | Hispanic vs NHW, all adults | 80 | 118 | 1,304 | 1,902 | −1.49 (−3.04 to 0.07) | 0.061 |
| Poor mental health days | NHB vs NHW, cancer | 16 | 25 | 366 | 531 | −4.72 (−8.95 to −0.49) | 0.029 |
| Poor mental health days | Hispanic vs NHW, cancer | 30 | 35 | 366 | 531 | −7.12 (−10.08 to −4.16) | <0.001 |

Within-Florida analyses compared non-Hispanic Black and Hispanic adults separately with non-Hispanic White adults. Adjusted DiD estimates are from linear regression incorporating the study-group-by-policy-period interaction and interview month-year fixed effects, with survey weights and adjustment for age, sex, education, and employment. Ns are unweighted and reflect the outcome-specific complete-case samples. Because the analyses were within a single state, state-level clustering and wild cluster bootstrap inference were not applicable. These subgroup analyses were exploratory and did not formally test differences between non-Hispanic Black and Hispanic adults. NHB, non-Hispanic Black; NHW, non-Hispanic White; CI, confidence interval.

**Table S8.** Wild cluster bootstrap inference for state-comparative difference-in-differences and difference-in-difference-in-differences models

| **Model** | **Adjusted estimate (cluster-robust 95% CI), days** | **State-clustered p value** | **Wild cluster bootstrap p value** |
| --- | --- | --- | --- |
| **Primary comparison: Florida vs Southern control states** |  |  |  |
| Overall, physical-health DiD | 1.11 (−0.55 to 2.76) | 0.228 | 0.175 |
| Cancer survivors, physical-health DiD | 4.97 (−0.59 to 10.52) | 0.118 | 0.137 |
| Adults without cancer, physical-health DiD | −0.55 (−1.79 to 0.70) | 0.415 | 0.609 |
| Physical-health DDD | 7.09 (3.63 to 10.54) | 0.004 | 0.032 |
| Overall, mental-health DiD | −0.76 (−2.52 to 1.00) | 0.423 | 0.507 |
| Cancer survivors, mental-health DiD | −3.46 (−5.94 to −0.97) | 0.026 | 0.159 |
| Adults without cancer, mental-health DiD | −0.76 (−1.91 to 0.40) | 0.235 | 0.322 |
| Mental-health DDD | −1.32 (−4.16 to 1.52) | 0.389 | 0.526 |
| **Race/ethnicity-stratified analyses: Florida vs Southern control states** |  |  |  |
| Non-Hispanic Black adults, physical-health DiD | 2.23 (0.96 to 3.51) | 0.009 | 0.048 |
| Hispanic adults, physical-health DiD | −0.56 (−6.94 to 5.83) | 0.868 | 0.890 |
| Non-Hispanic Black adults, mental-health DiD | −1.02 (−2.81 to 0.77) | 0.297 | 0.495 |
| Hispanic adults, mental-health DiD | −5.60 (−8.99 to −2.21) | 0.012 | 0.079 |
| Sensitivity analysis: Florida vs all other US comparison jurisdictions |  |  |  |
| Overall, physical-health DiD | 1.41 (0.87 to 1.94) | <0.001 | 0.312 |
| Cancer survivors, physical-health DiD | 8.26 (5.76 to 10.75) | <0.001 | 0.108 |
| Adults without cancer, physical-health DiD | −1.00 (−1.43 to −0.58) | <0.001 | 0.380 |
| Overall, mental-health DiD | −1.13 (−1.79 to −0.47) | 0.001 | 0.291 |
| Cancer survivors, mental-health DiD | −3.25 (−5.11 to −1.39) | 0.001 | 0.300 |
| Adults without cancer, mental-health DiD | −0.51 (−1.22 to 0.21) | 0.170 | 0.451 |

Adjusted estimates were obtained from models including state and interview month-year fixed effects, BRFSS survey weights, and the prespecified covariates. Wild cluster bootstrap p values used a null-restricted bootstrap with Webb six-point weights, 9,999 replications, and clustering by state; because Florida was the only treated state, both conventional and wild cluster bootstrap inference remain limited. DiD, difference-in-differences; DDD, difference-in-difference-in-differences; CI, confidence interval.

**Supplementary Figure 1.** Stratum-specific event studies for cancer survivors and non-cancer respondents.

Each point is the half-year-specific Florida x survey period difference-in-differences coefficient (in days) within the indicated stratum of the matched cohort, with January-June 2023 as the reference (open circle) and July 2023 excluded as a transition period. Model specification, weighting, covariates, fixed effects, and confidence intervals are as in the main figure (CR2/Satterthwaite cluster-robust, clustered by state); the 2021 survey periods are absent as described there. Joint pre-policy wild cluster bootstrap tests by panel: (A) cancer survivors, physical, p=0.331; (B) cancer survivors, mental, p=0.264; (C) non-cancer, physical, p=0.042; (D) non-cancer, mental, p=0.043. Y-axis scales differ across panels. Differential pre-policy coefficients were detected among non-cancer respondents; the corresponding dynamic triple-difference results are shown in the main figure.
